# Can Machines Learn Creativity Needs? An Approach Based on Matrix Completion

**DOI:** 10.1007/s40797-022-00200-8

**Published:** 2022-07-08

**Authors:** Giorgio Gnecco, Sara Landi, Massimo Riccaboni

**Affiliations:** IMT-School for Advanced Studies, Piazza S. Francesco 19, 55100 Lucca, Italy

**Keywords:** Creativity and soft skills, Counterfactual analysis, Matrix completion, Labor market, Automation, C44, C53, C63, J24, J44

## Abstract

Technological progress has been recently associated with a crowding-out of cognitive-skill intensive jobs in favour of jobs requiring soft skills, such as ones related to social intelligence, flexibility and creativity. The nature of soft skills makes them hardly replaceable by machine work and among subsets of soft skills, creativity is one of the hardest to define and codify. Therefore, creativity-intensive occupations have been shielded from automation. Given this framework, our study contributes to a nascent field on interdisciplinary research to predict the impact of artificial intelligence on work activities and future jobs using machine learning. In our work, we focus on creativity, starting from its possible definitions, then we get significant insights on creativity patterns and dynamics in the Italian labour market, using a machine learning approach. We make use of the INAPP-ISTAT Survey on Occupations (ICP), where we identify 25 skills associated with creativity. Then, we apply matrix completion—a machine learning technique which is often used by recommender systems—to predict the average importance levels of various creative skills for each profession, showing its excellent prediction capability for the specific problem. We also find that matrix completion typically underestimates the average importance levels of soft skills associated with creativity, especially in the case of professions belonging to the major group of legislators, senior officials and managers, as well as intellectual professionals. Conversely, overestimates are typically obtained for other professions, which may be associated with a higher risk of being automated.

## Introduction

Since the early 2000s, technological progress has been associated with a crowding-out of cognitive-skill intensive jobs in favour of jobs requiring soft skills, such as ones related to social intelligence, flexibility and creativity (Acemoglu and Autor 2011). The growth of occupations characterized by cognitive tasks has been sluggish both in terms of employment and wages (Acemoglu and Autor 2011; Beaudry et al. 2016; Deming 2017a). Moreover, the returns to cognitive skills have not risen (Castex and Dechter 2014).

Some authors suggest that this phenomenon could be ascribed to a progressive extension of the perimeter of the tasks at risk of automation toward those traditionally performed by high-skill workers (Levy and Murnane 2012; Brynjolfsson and McAfee 2014; Remus and Levy 2016; Autor 2014). Indeed, Deming (2017a) shows that it is especially the decrease in Science, Technology, Engineering and Mathematics (STEM) jobs, requiring high technical abilities but low social interactions, which has determined the shrinkage of high-skill/cognitive share of employment and wage bill. Instead, the cognitive occupations that have fared much better are those involving complex human interaction skills (related to communication, collaboration, teamwork, flexibility, creativity, etc.).

Those “social skills” are also referred to in the literature as “soft skills” or “non-cognitive skills” (Deming 2017b). According to Rainsbury et al. (2002), one can define soft skills as either behavioural, human, or interpersonal skills that an individual is required to possess with the aim of applying technical skills and knowledge in her/his workplace. Indeed, James and James (2004) claim that the term “soft skills” represents a novel way to refer to a set of either abilities or talents that a person can bring to the workplace. According to several authors, soft skills can be described as “micro social” skills, further categorized, e.g., as: (1) intrapersonal versus interpersonal skills; (2) personal versus social skills; (3) cognitive skills (Muzio et al. 2007). For what concerns the comparison between hard and soft skills, it is worth remarking that: (1) most people are able to distinguish between hard skills (e.g., working with equipment or software) and soft skills (e.g., interpersonal/intrapersonal focus) with quite relative ease; (2) there is quite a significant difference in learning for the two different cases of hard and soft skills; (3) the majority of positions inside an organization need—for a successful execution of the related work tasks—not only the acquisition of hard skills, but also a significant degree of proficiency in soft skills (Laker and Powell 2011).

This change of paradigm toward forms of organization of work characterized by an increased importance of social interaction skills has been noticed across several industries and occupations. Bessen et al. (2020) show that the adoption of automation technologies extends beyond manufacturing firms, while Katz and Margo (2014) suggest that the capability of combining the possibilities offered by automation and Information and Communication Technologies (ICTs) for product customization with creativity and communication skills in the relationship with customers is the key factor to explain and sustain the revival of artisanal jobs observed in the United States.

The rapidity and pervasiveness with which economic and organizational changes occur determine an increasing need in reading promptly and efficiently the evolutionary dynamics of all skills in general and soft skills in particular. Indeed, the nature of the soft skills makes them hardly replaceable by machine work, because they are a sort of tacit knowledge that human beings apply spontaneously and even unconsciously, built generation after generation, and not codifiable in “closed-form solutions”. Instead, cognitive tasks, though complex, are often much easier to codify.

Among subsets of soft skills, creativity is assuming increasing importance in shaping the future of employment. Easton and Djumalieva (2018) find that there is a strong demand for creativity in the online advertising job market and, following the work by Windsor and Bakhshi (2015), they are able to claim that creativity itself is consistently the most significant predictor for the chance of an occupation of growing (expressed as a percentage of the workforce), by the year 2030. A global study by Adobe, run in 2016, found that businesses which invest in creativity report an increase in employee productivity (78%) and customer satisfaction (80%). Silvestri et al. (2018) found that creative inventions (i.e., unconventional patents) are associated with a higher technological impact and market value during recessions.

As the European Union recently stated in the Entrepreneurship Competence Framework, creativity refers to a set of soft skills characterized by a strong strategic component, and goes together with other skills such as learning, innovation, project management, proactivity, and adaptability. Hence, focusing on such skills allows one to reflect upon the possibilities that workers have to be active parts inside firms. Creativity is, in fact, a pillar of the varied concept of entrepreneurship, and represents one of the fifteen competences that articulate the entrepreneurship framework (Venckutė et al. 2020). According to the empirical findings obtained by the European Working Condition Surveys (Eurofound 2005, 2015), every second workplace involves some form of “creative work”, which is not significantly threatened by automation, whereas every fourth worker carries out “routine” tasks that are likely to be automated in the near future. Moreover, the EU National Reports on the labor market soft skills (Pérez et al. 2020) have recently addressed some relevant issues for the Italian labor market that require further investigation and policy maker attention: in Italy, job quality is often low and there is a high skill mismatch, implying that the future occupational trends will see an increase of low and medium level jobs. This evident skill emergency and the consequent job instability makes it crucial to understand which are the strongest needs in the soft skill endowments of Italian workers. It is worth remarking that the Italian labor market suffered a huge crisis after the Great Recession in 2008–2011, followed by a sovereign debt crisis that worsened an already unstable situation. Moreover, starting from the early 2000s, Italy’s labor market has undergone substantial reforms aimed at improving flexibility of job contracts. Still, one observes high unemployment, low productivity, high skill mismatch and a trend that favors low-skilled over high-skilled jobs (Hoffman et al. 2021). All these characteristics, combined with the richness of data available, makes Italy a perfect candidate for our analysis, which is described in the following.

In this paper, we exploit similarities in the Italian occupational structure and implement matrix completion—a recently developed machine learning technique which is often used by recommender systems (Hastie et al. 2015)—in order to predict the average importance levels of creative soft skills (i.e., soft skills associated with creativity) employed in each occupation and to identify peculiarities of such occupations and such skills. For the purpose of our analysis, we describe creativity along four dimensions and we identify in our data 25 skills associated with creativity. We then formulate a matrix completion optimization problem for the prediction of the average importance levels of creative soft skills (or, in short, for creativity levels prediction), and we solve it through a state-of-the-art algorithm (the Soft Impute algorithm). In more detail, the objective of our analysis is twofold: on the one hand, we investigate if matrix completion is able to predict with good accuracy elements belonging to selected columns (associated with skills) of a suitable occupation matrix^1^ (which is derived from a recent survey), when such elements are artificially obscured (i.e., when only a portion of that matrix is made available as input to matrix completion); on the other hand, after providing a positive answer to the first question, we employ matrix completion to compare expected creativity levels (i.e. the elements of the original matrix that are predicted by matrix completion), with the associated actual creativity levels (i.e., with the corresponding elements of the original matrix, in the same positions that have been previously hidden artificially to matrix completion). This is done with the aim of identifying “outlier” professions whose “creativity needs” (as encoded by elements belonging to the selected columns of the matrix) are, respectively, much larger/much smaller than expected (according to their data-driven predictions, which are produced as outputs by matrix completion).

In other words, we apply matrix completion to identify a counterfactual value (or expected value, based on the information coming from the observed portion of the matrix) for the average importance level of each creative skill across professions. Then we get from the results of our analysis that such counterfactual value is typically in good agreement with the actual average importance level. However, for some professions, the observed level deviates positively/negatively from the counterfactual (i.e., the counterfactual provides an underestimate/overestimate), hence such professions are associated with a surplus/deficit in creativity levels. In this regard, the methodological approach used in this work resembles the one employed in the analysis of total factor productivity, in which a set of actual outputs is compared versus the corresponding set of predicted outputs according to given inputs and a specific production function.^2^ In this case, the emergence of outliers may be explained, e.g., by the absence of relevant inputs in the model or by its lack of additional nonlinear terms.

To the best of the authors’ knowledge, this is the first article in which the matrix completion technique mentioned above is applied to analyze workers’ skills in a whole economy, with the aim of predicting suitable elements of an occupation matrix as functions of the other elements of that matrix. Our analysis shows that: (a) matrix completion demonstrates an excellent prediction capability on the specific dataset, making it meaningful to analyze cases for which the actual average importance levels of skills associated with creativity is significantly larger/smaller than the predicted one. Indeed, given the extremely good fit typically obtained, a large error in the prediction of the average importance levels of skills associated with creativity for a specific profession may be imputed to peculiarities of that profession, which make it less similar to other professions in the dataset and, as a consequence, more/less “hungry” of creative skills with respect to those professions^3^; (b) soft skills associated with creativity are not neatly separated among intellectual and technical workers; (c) some professions show a surplus of creativity with respect to its level predicted by their counterfactuals. In this case, their risk of being replaced by machines is deemed to be low; (d) on the other hand, we get a deficit of creativity for other professions. In this case, the risk for such professions of being replaced by artificial intelligence techniques is larger. In order to reduce this risk, training on creative soft skills might be tailored on these specific professions.

The paper is structured as follows. Section 2 reports a review of related literature. Section 3 describes the dataset used in our analysis. Section 4 summarizes the machine learning approach adopted in the work (more technical details about it are reported in the Appendix). Section 5 provides the main results of the analysis. Section 6 concludes the article with a discussion.

## Related Literature

Our work builds on four main streams of existing literature dealing respectively with: (1) the relationship between ICT and soft skills; (2) skill relatedness, (3) creativity; (4) matrix completion. In the work, these issues are connected as follows. Our analysis allows the assessment of patterns and peculiarities of creative soft skills in the Italian labor market, as the endowments and formation of creativity among Italian workers could mitigate the negative effects of digitalization and job automation on low-skilled professions. More specifically, our analysis relies on the application of matrix completion to an occupation matrix whose entries represent, e.g., average importance levels of various soft skills (including ones associated with creativity) in different jobs. As detailed in the work, such an occupation matrix is quite well approximated by a low-rank matrix (an essential assumption for its successful application^4^), which could be justified by the presence of skill relatedness across industries.

*(1) Relationship between ICT and soft skills*

Numerous theoretical and empirical studies investigate the effect of technological progress on jobs, paying particular attention to the growth in importance of social skills in the labour market as an increased interest in social interactions skills has been noticed across all industries and occupations. The diffusion of ICT, and more broadly of “digital technologies”, has in fact led to significant transformations in several organizational aspects of consumption models, firms, industries, labour activities, and production processes. In the seminal paper by Autor et al. (2003), the authors claim that digitalization increases the possibility of making tasks automated, when these are characterized by a high degree of routineness. This is due to the fact that routine tasks are typically more easily codified, as compared to non-routine tasks.

In a very recent work, Bachmann et al. (2022) studied the impact of robot adoption on workers’ transitions (from employment to unemployment and viceversa) in Europe. They found small negative effects on job separations and small positive effects on job findings, with these effects being stronger for intensive routine manual or routine cognitive tasks occupations. In a similar vein, Domini et al. (2021b) found that automation spikes are associated with an increase in firms’ simultaneous net employment growth rate, and that these can be jointly explained by a larger hiring rate and a smaller separation rate, while Harrigan et al. (2016) highlighted how automation polarized French labor market in France. According to the authors of the latter work, such a polarization was mostly driven by changes in the composition of firms in each industry. Specifically, they found that “techies” (i.e., technology-related occupations) were an important force driving aggregate polarization in France at the time, as firms with more techies grew faster. Moreover, by focusing on French data, Domini et al. (2021a) found that the adoption of automation- or AI-related capital goods was followed by wage increase (1%) 3 years after these events, and that this increase was due to the hiring of new workers. It is worth mentioning that the concept of routinitity of a task does not apply only to the cases of low skilled and unqualified (manual) functions, but also to those cognitive tasks that are carried out principally by managers and occupational workers. Therefore, one can distinguish between routine and non-routine tasks (e.g., in the case of manual tasks): while the latter exhibit a complementary relationship to digital technologies, cognitive routine tasks (which are typical of clerical workers and administrative jobs) are potentially highly exposed to automation, due to the introduction of digital technologies (Autor et al. 2003).^5^ To conclude, the literature on the task-based approach is rapidly expanding, and many papers have appeared recently, including recent contributions related to Italy as a case study (see, e.g., Bonacini et al. 2021; Carbonero and Scicchitano 2021; Caselli et al. 2021; Cetrulo et al. 2020; Esposito and Scicchitano 2022; Vannutelli et al. 2021).

*(2) Skill relatedness*

The term “skills” includes a wide range of qualitatively different capabilities that could encompass a huge variety of more specific competences, used in particular occupations and industries (Fitjar and Timmermans 2017). This implies that, to some extent, skills are industry-specific and the stronger the relatedness between industries, the smaller human capital destruction occurring when labor flows from one industry to another (Galetti et al. 2021).

Skills acquired in a specific industry, in fact, are often useful also in the context of other industries. Given the extremely relevant role assumed by human capital in a firm’s strategic asset stocks, a firm is expected to concentrate its efforts of diversification in areas that require skills which are already part of the background of its current workforce. In their pioneering work, Neffke and Henning (2013) proposed a quantitative way to define the similarity of industries’ human capital or skill requirements (i.e., the industries’ skill relatedness), by taking into account information associated with cross-industry labor flows. They showed that firms are significantly more likely to achieve diversification into industries that are linked to the firms’ core activities in terms of some skill-relatedness measure than into industries that do not present such ties.

*(3) Creativity*

The central importance of creativity as one of the main engines of scientific and social progress has been recently witnessed by a large number of related research efforts, all having the common goal of understanding its origin and nature. Among the various approaches followed by researchers to understand creativity, it is worth mentioning explanations of creativity from several points of view related, e.g., to mysticism, pragmatism, psychodynamics, psychometrics, cognition, socio-personality, and also multidisciplinary approaches (Sternberg and Lubart 1999). These different perspectives have provided valuable insights into creativity, but they have also been seen as having hindered some serious psychological research on this research topic. The diffused focus of research has also limited any possible conceptual agreement on what creativity exactly is. Indeed, in the literature, creativity has been variously described, e.g., as a process, product or personal trait. Some researchers have interpreted creativity as a staged process (e.g., Amabile 1983; Basadur et al. 1982), or as a cognitive process associated with the production of diverging ideas (De Bono 1992; West and Farr 1990).

The literature review conducted by Walia (2019) shows the existence of quite a general agreement among scholars about the fact that the concept of creativity is associated with the production of original and useful ideas and products (Mumford 2003). Runco and Jaeger (2012) observed that the standard definition of creativity (which requires the presence of some elements of originality and effectiveness) has quite a long history in the related literature. Hennessey and Amabile (2010) argued that the requirement to implement a creative idea can be considered as the origin of the innovation process. Most recent research on creativity focused on novelty and usefulness of ideas as its peculiar features (Mumford 2003). More in detail, novelty refers to originality, i.e., to the production of something which can be considered as new, whereas usefulness is related to the suitability of an idea for solving a specific problem under investigation (Amabile and Pratt 2016; Hennessey and Amabile 2010). Some proposals of a definition of creativity include more stringent criteria, such as high quality level (Sternberg and Lubart 1995), absence of conformity (Niu and Sternberg 2002), surprise (Boden 2004), non-obviousness (Simonton 2012), and aestheticism accompanied by authenticity (Kharkhurin 2014).

Mumford et al. (2002) reported two different groups of processes, both related to creative work: (a) activities that lead to the generation of a novel idea; (b) activities that are required to implement such ideas (implementation). As an example, the identification of a still unsolved problem (conceptualization) certainly requires creativity and, according to Csikszentmihalyi (1988), could be even interpreted itself as a creative task. Taking simultaneously into consideration various heterogeneous elements that may jointly interact during the related processes of conceptualization, ideation, and implementation, creativity is sometimes modeled in the literature as a socio-psychological phenomenon (Amabile and Pillemer 2012), i.e., as a phenomenon in which several distinct characteristics of an individual interact, e.g., with her/his culture and environment (Lebuda and Csikszentmihalyi 2018). Even though the acts of creation may be considered to be individualistic at a first sight, their origin can be actually social (Glăveanu 2013). Finally, at the level of an individual, creativity could be interpreted as a construct of one’s imagination (Lindqvist 2003); nevertheless, such a process is actually shaped by the creator’s everyday interactions with her/his specific environment, taking into account, e.g., of the specific cultural, historical, or ideological context (Thibodeaux 2014).

A possible analytical definition of creativity comes from the seminal work of Edward De Bono and Efrem Zimbalist (De Bono and Zimbalist 1970). The authors defined lateral thinking as a set of different processes associated with a deliberate, systematic way of thinking in a creative way, i.e., related to an innovative way of thinking that is able to occur in a repeatable manner. According to their pioneering research in the field, lateral thinking is strictly related to creativity and can be described along four dimensions: (1) fluidity, as the ability of a subject to give the highest possible number of answers to a certain question; (2) flexibility, as the number of categories to which one can bring back these questions; (3) originality, as the ability of expressing new and innovative ideas; (4) processing, as the ability of realizing concretely one’s ideas. Following this path, many researchers defined creativity as a set of strategic soft skills, which allow workers to find result-oriented solutions to open problems (Tucciarelli 2014; Ciappei and Cinque 2014; Pellerey 2016). Therefore, creativity goes hand in hand with other competences such as learning, innovation, project management, proactivity and adaptability to change.

Teamwork and creativity require a set of abilities that have been defined in the related literature as communication and mutual understanding of each other’s preferences, motivations and comparative advantages, i.e., a kind of ability that has been conceptualized within the theory of mind (Premack and Woodruff 1978; Baron-Cohen 2000; Camerer et al. 2005). Starting from the work by Autor et al. (2003), tasks requiring creativity have been considered problematic to computerize. Indeed, recombining existing features to create new ideas is easily automatable, but recognizing whether these new combinations make sense and are valuable requires encoding an objective set of creative values (Frigotto and Riccaboni 2011). Moreover, Windsor and Bakhshi (2015) and Frey and Osborne (2017) suggested that the creative process is hard to codify and, therefore, that creativity-intensive occupations have been shielded from automation. This remark extends to other soft skills different from the ones associated with creativity.

*(4) Matrix completion*

We conclude this literature review by addressing its last point, i.e., the application of machine learning to the specific context of the analysis of professions (this is only one of the many applications machine learning has found in the last years). Recent works devoted to this specific application are, e.g., Cockburn et al. (2018), Goldfarb et al. (2020), Iansiti and Lakhani (2020), and Lanzolla et al. (2021). In this framework, as already mentioned, in the present work creativity levels of different professions—measured through a recent survey, and collected in a suitable occupation matrix—are analyzed based on a recently developed machine learning technique, which is called matrix completion (Hastie et al. 2015). The choice of matrix completion for our analysis is due to the fact that this technique is often used by recommender systems (Ricci et al. 2011) to infer users’ preferences, then suggest items to them, based on the inferred preferences. This is motivated by the ability of matrix completion to capture possibly hidden relationships among elements of a rating matrix (e.g., in the case of a movie-rating matrix, to capture the similarity between movies, or between users’ preferences). A similar argument can be applied in the case of our occupation matrix. In this application, a low/high risk of automation for a profession may be associated with an underestimate/overestimate by a state-of-the-art machine learning algorithm of the creativity needs of that profession, based on a suitable training set. In the present context of the analysis of professions, it has to be mentioned that recommender systems are adopted, e.g., for job recommendation (Al-Otaibi and Ykhlef 2012). In the economic context, matrix completion can be exploited, e.g., to construct synthetic controls (Amjad et al. 2018), for their possible successive use in counterfactual analysis (Athey et al. 2021). Another possible application is in recommender systems for education. Variations of matrix completion have also been developed in the literature to discover relationships among different databases, assuming that each of them is modeled by a matrix (Bouchard et al. 2013). In the present work, matrix completion is applied for two purposes: in order to assess its prediction capability of the creativity needs of different professions, and with the aim of generating counterfactuals. It has to be remarked, indeed, that in the context of job market analysis, matrix completion has not been applied yet to define theoretical counterfactuals of expectations of average importance levels of skills. Such counterfactuals can be useful, e.g., to identify specific variations in the creativity needs of the various professions.

## Data

ICP (*Indagine Campionaria sulle Professioni*, which can be translated into English as *Sample Survey on Professions*) is a survey on workers, promoted by the Italian National Institute of Statistics (ISTAT) and by the National Institute for Public Policies Analysis (INAPP). It was last run in 2013 by INAPP (INAPP 2013) on nearly 16.000 Italian workers in about 800 occupations, according to the 5-digit CP2011 classification (which is the Italian equivalent of the ISCO-08 ILO’s classification).^6^ The survey reports more than 400 variables related to skills, attitudes and tasks, and is based on 20 workers on average per each Italian occupation. The sample is stratified so as to make it possible to achieve a high level of representativeness with respect, e.g., to firm size, geographical domain (at the level of macro-regions), occupation, and sector. The ICP survey investigates several characteristics of the occupations, based on a particularly rich and articulated questionnaire, which is structured in six main sections. These are the expressions of a content model able to provide simultaneously information from both a job-oriented and a worker-oriented perspective: (1) worker characteristics (enduring abilities and work style of workers); (2) worker requirements (skills and education); (3) occupational requirements (organisational and work context); (4) experience requirements (training, cross functional skills); (5) workforce characteristics (labour market information); and (6) occupation-specific information (generalised activities and work context).^7^ In doing this, several descriptors are considered by the survey, making it possible to discriminate, for instance, between inner individual abilities and competences acquired for the specific job.

Information in the survey is reported by entrepreneurs and Human Resources (HR) responsibles. Respondents are asked to report the skill needs of their employed workforce to be satisfied in the short run, as future trends and new training needs are mainly related to competences and soft skills. For almost every question, two rating scales are provided: e.g., in the case of a skill, participants are asked to assess the importance level of that skill in their job and, provided their answer exceeds a suitable threshold, the intensity level in the use of such skill.

The ICP directly asks workers, instead of experts, to answer the questionnaire. In this way, the focus of the survey is given on the viewpoint of those who exercise the analyzed daily occupational activities, since they are able to provide a direct and concrete assessment of the importance and the intensity level of use of certain characteristics, which, depending on the answer, may turn out to be either essential or inessential to carry out one’s job (Barbieri et al. 2022).

The ICP is a unique source of information on the work content. In fact it is the only survey replicating the US O*NET, the most comprehensive structure containing qualitative and quantitative information on job tasks, work context and workplaces at a very detailed level. As well as the US O*NET, also the Italian ICP focuses on occupations, so variables are built based on worker-level surveys and a post-survey validation made by focus groups. It is worth noticing that tasks and skills are specific to the Italian economy. So, the ICP can be used to define the structure of the labour market, the level of technology and the industrial relations, characterizing the Italian economy. Overall, the ICP dataset has a double advantage: it is accurate, granular, and rich, and most importantly, it is specific to the Italian productive system. No international crosswalk (based for instance on US data) is needed. Thus, one possibly avoids biases arising when information referring, e.g., to the US occupational structure are linked to labour market data referring to different economies such as the European ones. More information on that can be found in Bonacini et al. (2021) and Vannutelli et al. (2021).

The ICP follows the Classification of Occupations (CP2011) produced by ISTAT in 2011 and, along this line, it divides jobs into eight major groups:

1. Legislators, managers and senior officials;
2. Intellectual, scientific and highly specialized professionals;
3. Technicians and associate professionals;
4. Clerical support workers;
5. Service and sales workers;
6. Artisans, specialized and agricultural workers;
7. Plant and machine operators and assemblers;
8. Elementary occupations.

Table 1 reports some descriptive statistics of the sectors in our available sample, related to the ICP survey performed in 2013. As the sample is representative of the Italian occupational structure, 4% of the observations belong to the primary sector (agriculture and farming), while the majority of workers (65%) is employed in one among services, other services, healthcare system, and cultural industry. More specifically, in the manufacturing sector we included those jobs that are specifically associated with goods transformation (i.e., related to metallurgical, siderurgical, chemical, pharmaceutical, mechanical, automotive, aerospatial, railway, electronic, food, textile industries) while the term “other industries” refers to all the other industrial activities, such as agricultural industry, retail and distribution, energetic industry, transports and logistics, constructions. In the services sector, we included all those activities related to accommodation and catering, tourism, education, finance and banking, professional and scientific professions, real estate, public administration; while the term “other services” is related to services to the person and associations. Given the large availability of information included in the ICP, we decided to consider in our successive analysis not only skills, but also those variables that refer to working attitudes and styles as well as generalized work activities. We ended up identifying 25 items in the ICP survey as being associated with creativity, as highlighted in Table 2. Our choice of the set of 25 items was based on the opinion of an expert from INAPP (Riccaboni et al. 2021), taking into account the items available in the survey and the various definitions of creativity reported in the literature review of Sect. 2. Our resulting occupation matrix, coming from the ICP survey, is given by $$m=796$$ rows which refer to professional units and $$ n=255$$ columns, of which 55 denote skills (25 directly associated with creativity, and the other 30 not directly associated with it), whereas the other 200 columns refer to competences, working conditions, and working styles. Each entry in position (*i*, *j*) represents the average importance level^8^ (expressed as a percentage between $$0\%$$ and $$100\%$$) of skill/competence/working condition/working style *j* by worker type *i*. The 25 columns associated with the creative skills, on which our successive analysis is focused, form a subset *J* of columns of our occupation matrix.

**Table 1:** Descriptive statistics of the sectors covered by the ICP dataset (INAPP 2013)

Variables	n. of professions	% of professions
Primary sector	34	4
Manufacturing sector	161	20
Other industries	97	12
Service sector	315	40
Other services	112	14
Healthcare system	40	5
Cultural industry	37	5

**Table 2:** Soft skills associated with creativity in the ICP dataset

Indicator	Skill	ICP item code	Acronym
	Solving complex problems	C17A	COMPL
	Solving unexpected problems	C27A	UNEX
	Listening actively	C2A	LIST
*Fluidity—Flexibility*	Adaptability	F10	AD
	Service orientation	C16A	SERV
	Classification	D11A	CLASS
	Comprehension	D16A	COMPR
	Critical sense	C7A	CRIT
*Originality—Processing*	Analytical skills	C18A	ANSK
	Decision making	C31A	DMAK
	Originality	D6A	OR
	Production of ideas	D5A	IDEAS
	Learning strategies	C9A	LEAST
	Active learning	C8A	ALEARN
*Learning—Innovation*	Teaching	C15A	TEACH
	Creative thinking	G11A	CREAT
	Innovation	F15	INN
	Persuading	C13A	PERS
	Understanding others	C11A	UNDOTH
	Negotiating	C14A	NEG
*Planning—Proactivity*	Time management	C32A	TIME
	Financial resources management	C33A	FRM
	Material resources management	C34A	MRM
	Human resources management	C35A	HRM
	Coordination with others	C12A	COORD

In Table 3 we report the (empirical) mean and standard deviation (with respect to the professions) for the 25 creative soft skills we consider in our analysis. The values shown are percentages, meaning that workers answer on a scale from 0 to 100 to the question “How important is this soft skill in performing your job daily?”. We can clearly see that all these skills are relevant for the entire population, in particular, listening actively, adaptability and coordination with others are required the most.

**Table 3:** Mean and standard deviation for the 25 creative soft skills

Skill	Mean	Std dev
Listening actively	71.74	13.23
Adaptability	69.53	10.32
Coordination with others	64.57	13.44
Time management	63.94	12.79
Critical sense	59.50	19.24
Active learning	56.63	17.84
Innovation	56.10	18.40
Solving complex problems	55.82	18.80
Service orientation	54.84	15.54
Teaching	51.09	18.11
Understanding others	50.48	18.22
Comprehension	50.43	13.73
Decision making	47.61	18.84
Classification	46.69	15.22
Negotiating	46.65	18.07
Originality	46.60	20.99
Analytical skills	46.42	15.57
Material resources management	44.88	15.01
Creative thinking	44.17	22.68
Learning strategies	43.96	18.86
Production of ideas	41.77	16.57
Human resources management	39.89	21.25
Financial resources management	39.32	24.25
Solving unexpected problems	39.09	18.15
Persuading	38.46	16.73

## Matrix Completion

In the following, we apply Matrix Completion (MC) to predict average importance levels of skills associated with creativity (or, shortly, creativity levels) for the various professions in our occupation matrix, exploiting the similarity patterns detected automatically by that machine learning technique from its application to the specific dataset.

MC can be defined as the task of filling in the missing entries of a partially observed matrix (Recht 2011). One well-known example of such a matrix is the rating matrix in a recommender system representing users’ tastes on products (e.g., movies). Given a subset of elements of a rating matrix, in which each entry in position (*i*, *j*) represents the rating of movie *j* by customer *i*, if customer *i* has watched movie *j* and is otherwise missing, one would like to predict the other entries of the matrix in order to make good recommendations to customers on which movie to watch next. In order to do that, one exploits similarities between users (rows) and between products (columns), as one expects that users assigning the same or similar ratings on different products will share similar interests on new products, resulting in a low-rank structure of the rating matrix. It is worth remarking that, in this application to recommender systems, MC is used with the final goal of predicting the values assumed by a subset of entries of the rating matrix for which no ground truth is available at the time of prediction.^9^ Still, in order to perform a validation of an MC algorithm (for instance, with the aim of selecting an optimal value for one hyperparameter of its associated optimization problem), the MC predictions are typically compared with the ground truth coming from another subset of entries that can be actually observed, but that are artificially obscured to the MC algorithm. In this way, one has the possibility of assessing in a fair way the prediction capability of the MC algorithm. A similar use is made in our present application (for which the occupation matrix is actually known as a whole). Moreover, one reason for which we expected MC to be successful for our application is that, in a similar way as in its use for recommender systems, our occupation matrix turned out to be quite well approximated by a low-rank matrix (see the next paragraphs for details). A possible explanation for this is that workers in different industries acquire specific skills, and when they move to other professions, they apply such skills in the new context. As a consequence, jobs in different industries (i.e., rows in our occupation matrix) may share similar importance and intensity levels of soft skills.

In order to apply MC to our occupation matrix (or, for a successive analysis, to its suitable submatrix, see Sect. 5.1), we generate artificially partially observed matrices from it, by artificially obscuring randomly 10%, 25% and 50% of the entries in the 25 columns associated with the creative skills, focusing each time on the prediction capability of matrix completion on each single row (occupation). The procedure is repeated several times (details are in the Appendix). This sampling procedure is justified by the fact that we want to apply MC to predict entries of the occupation matrix that are related to creativity (i.e., that belong to one of the 25 selected columns). The three choices $10\%$, $25\%$ and $50\%$ are made in order to assess the accuracy (and robustness) of MC for quite different percentages of obscured entries. Finally, the motivation itself behind artificially obscuring known elements of the occupation matrix is that, in this way, it is possible to validate/test the predictions obtained by MC (i.e., a ground truth is available for comparison purposes).

In summary, we consider the following nuclear-norm regularized MC optimization problem:

1 $$\begin{aligned} \underset{\mathbf{Z} \in {\mathbb {R}}^{m \times n}}{\mathrm{minimize}} \left( \frac{1}{2} \sum _{(i,j) \in \Omega ^{\mathrm{tr}}} \left( M_{i,j}-Z_{i,j} \right) ^2 + \uplambda \Vert \mathbf{Z}\Vert _*\right) , \end{aligned}$$

where $\Omega ^{\mathrm{tr}}$ is a training set of positions (*i*, *j*) corresponding to the known entries of the partially observed matrix $\mathbf{M} \in {\mathbb {R}}^{m \times n}$, $\mathbf{Z} \in {\mathbb {R}}^{m \times n}$ is the completed matrix, $\Vert \mathbf{Z}\Vert _*$ is its nuclear norm, and $\uplambda \ge 0$ is a regularization constant. Then, we solve it by applying the Soft Impute algorithm (Mazumder et al. 2009). This is proved therein to converge to an optimal solution of the optimization problem (1). The optimization problem itself is solved by the Soft Impute algorithm several times, for different choices of the set of obscured entries. For each such repetition, the best value of $\uplambda$ is found by minimizing a suitable error on a validation subset of missing entries, whereas the final performance is evaluated on the remaining test set of other missing entries. Technical details about the choice of the various training/validation/test sets and of the regularization parameter of the optimization problem (1) are reported in the Appendix. Additional details about the optimization problem (1) and the Soft Impute algorithm are reported also in Metulini et al. (2022).

**Fig. 1:**
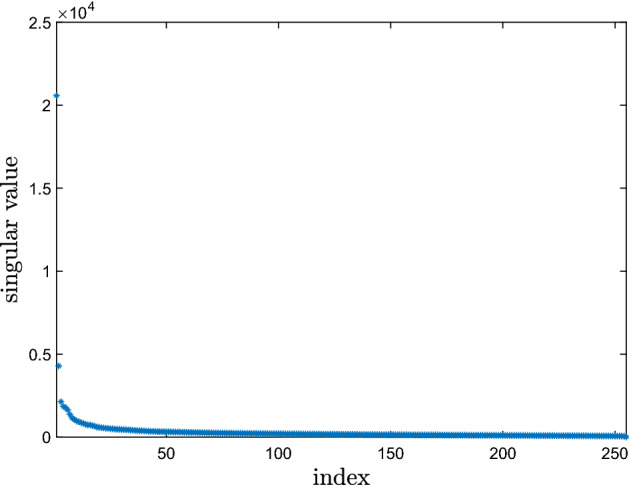
Distribution of the singular values of the occupation matrix considered in the analysis

As a preliminary check for the applicability of MC, we compute the singular value decomposition of our occupation matrix. Figure 1 shows that its singular values decay quite fast to 0, hence that the matrix can be well approximated by a low-rank one. As reported in the Appendix, this is a necessary condition for an effective application of MC, which is satisfied by the dataset under analysis.

**Fig. 2:**
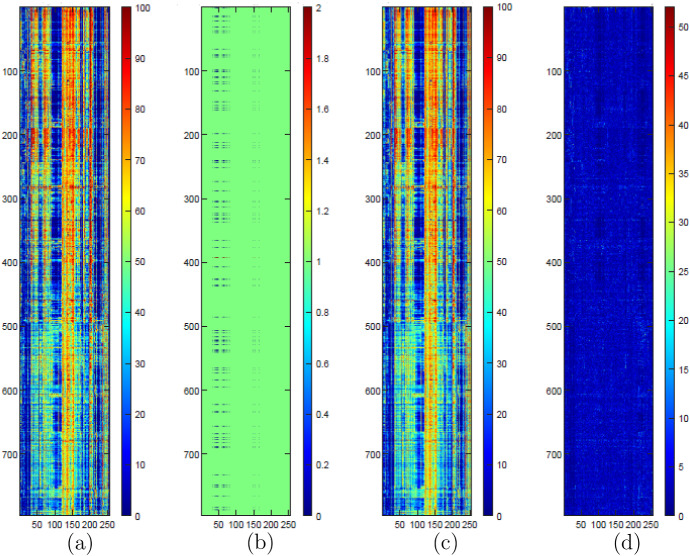
Visual representation of **a** the original matrix, **b** the locations of its observed (green) and missing entries (blue: validation; red: test), **c** the reconstructed matrix for a specific repetition, and **d** the absolute value of the prediction error for the same repetition (color figure online)

In order to better illustrate the approach used in our analysis, we consider a specific instance of the optimization problem (1), then we visualize its results. Figure 2 shows the original matrix, the locations of the observed and the missing entries in the original matrix (where the green cells denote the elements in the training set, the blue cells refer to the validation set and the red ones are related to the test set), the reconstructed matrix, and the error in absolute value for each cell. As the second subfigure shows, all the obscured entries are at the intersection between specific rows (one of which is associated with the test set) and the set of columns associated with creativity.

**Fig. 3:**
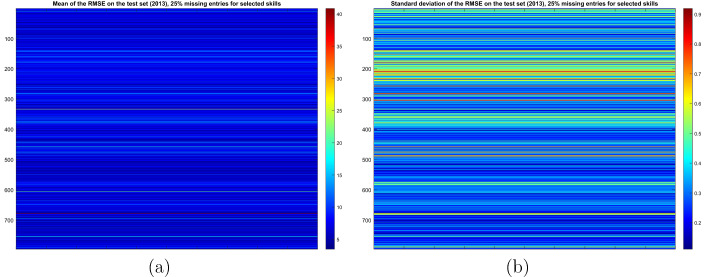
**a** Mean and **b** standard deviation of the RMSE of prediction on the test set per profession, for a specific choice of the percentage of missing entries in the columns associated with creativity (color figure online)

Then, Fig. 3 presents a visual representation of the (empirical) mean and standard deviation of the Root Mean Square Error (RMSE) of MC prediction on the test set (see the Appendix for details on its definition) per profession, where each row (profession) refers to the mean and standard deviation computed with respect to the repetitions having as test set elements belonging only to that specific row of the original matrix. As the figure shows, both the mean and standard deviation per profession are typically small (taking into account that the entries of the original matrix are numbers between 0 and 100).

**Fig. 4:**
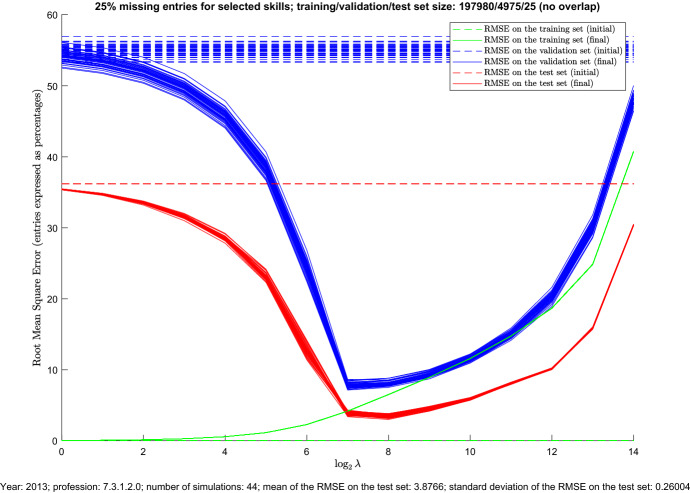
RMSE of prediction on the training, validation and test sets as functions of the regularization parameter $\uplambda$, for all the repetitions associated with the same test set related to a specific profession (color figure online)

**Fig. 5:**
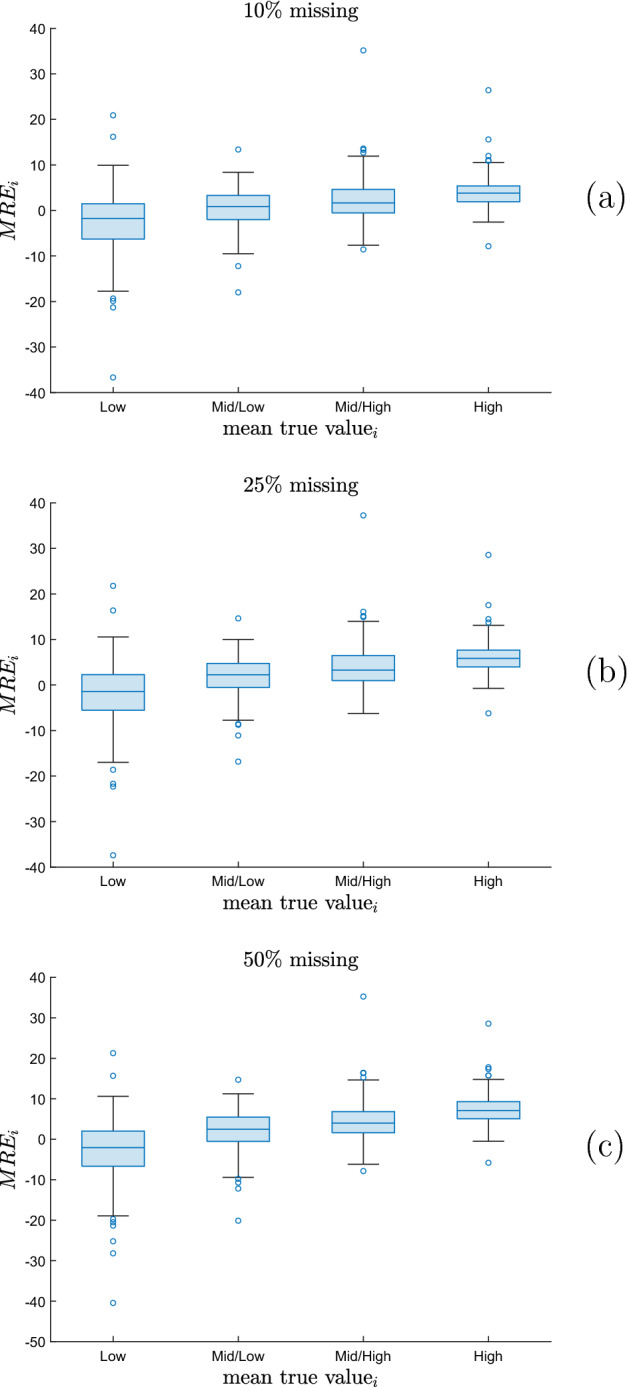
Boxplots of $MRE_i$ for 4 subsets of professions *i* corresponding to the 4 quartiles of the average true value $\frac{1}{25}\sum _{ \in J} true_{i,j}$ of the skill importance levels, for the skills directly associated with creativity: **a** $10\%$ missing values in the selected columns; **b** $25\%$ missing values in the selected columns; **c** $50\%$ missing values in the selected columns

Figure 4 reports more detailed results for all the repetitions for which the elements of the test set refer to a specific profession. In particular, the figure shows how the RMSE of MC prediction (evaluated, respectively, on the training set, validation set, and test set) changes as a function of the regularization parameter $\uplambda$. The sizes of the three sets are also reported in the figure. As the figure reveals, the optimal value of $\uplambda$ (which is computed based on the RMSE of MC prediction on the validation set) is associated with a quite small RMSE of MC prediction for both the validation and test sets. Moreover, for each of the two cases, the variability of the RMSE curve with respect to changing the training set (i.e., performing another repetition of the analysis, for the same choice of the test set) is very small, whereas the variability of the RMSE curve of MC prediction for the training set itself is negligible. It is worth mentioning that, for the specific case reported in Fig. 4, the performance of the method on the test set is slightly better than the one on the validation set. This is likely due to the fact that the elements of the two sets are sampled from different portions of the occupation matrix. Moreover, the test set has a much smaller cardinality (since its elements come from a specific row of the occupation matrix). In any case, the behaviour of the RMSE curve as a function of the regularization parameter $\uplambda$ is similar for both sets, being also the minima of these two curves achieved for almost the same values of $\uplambda$. Although Figs. 3 and 4 refer only to the percentage $25\%$ of missing entries in the selected columns associated with creativity, similar results have been obtained for the other two percentages.

Finally, Fig. 5 reports, for each of the three percentages of missing values in the 25 selected columns, the boxplots of the Mean Relative Error per profession, denoted as $MRE_i$ and defined later in Eq. (2), for 4 subsets of professions *i*. Such subsets correspond to the 4 quartiles of the average true value $\frac{1}{25}\sum _{j \in J} true_{i,j}$, where *J* is the set of the 25 skills directly associated with creativity, in such a way that the average is limited to such skills. The figure clearly shows that the surplus tends to be larger for the professions with a larger average true value of creative skill levels. This holds for each of the three percentages of missing values.

## Results

For each profession *i*, we predict (via the MC estimate $predicted_{i,j,r}$) the missing entry $true_{i,j}$ of each of the 25 skills $j \in J$ associated with creativity, in each repetition *r* belonging to the set of $r_i$ repetitions in which such elements of row *i* are in the test set. Then, we compute the associated Mean Relative Error, defined as follows (and expressed as a percentage):

2 $$\begin{aligned} MRE_i=\frac{1}{r_i} \sum _{r=1}^{r_i} \left[ \frac{\sum _{j \in J} (true_{i,j}-predicted_{i,j,r})}{\sum _{j \in J} true_{i,j}} \right] \times 100 \end{aligned}$$

To avoid burdening the notation, the dependence on the percentage of missing elements in the 25 selected columns is not reported in Eq. (2). We say that for a specific profession *i* there is a surplus (deficit) of creativity when $MRE_i>0$ (when $MRE_i<0$).

**Fig. 6:**
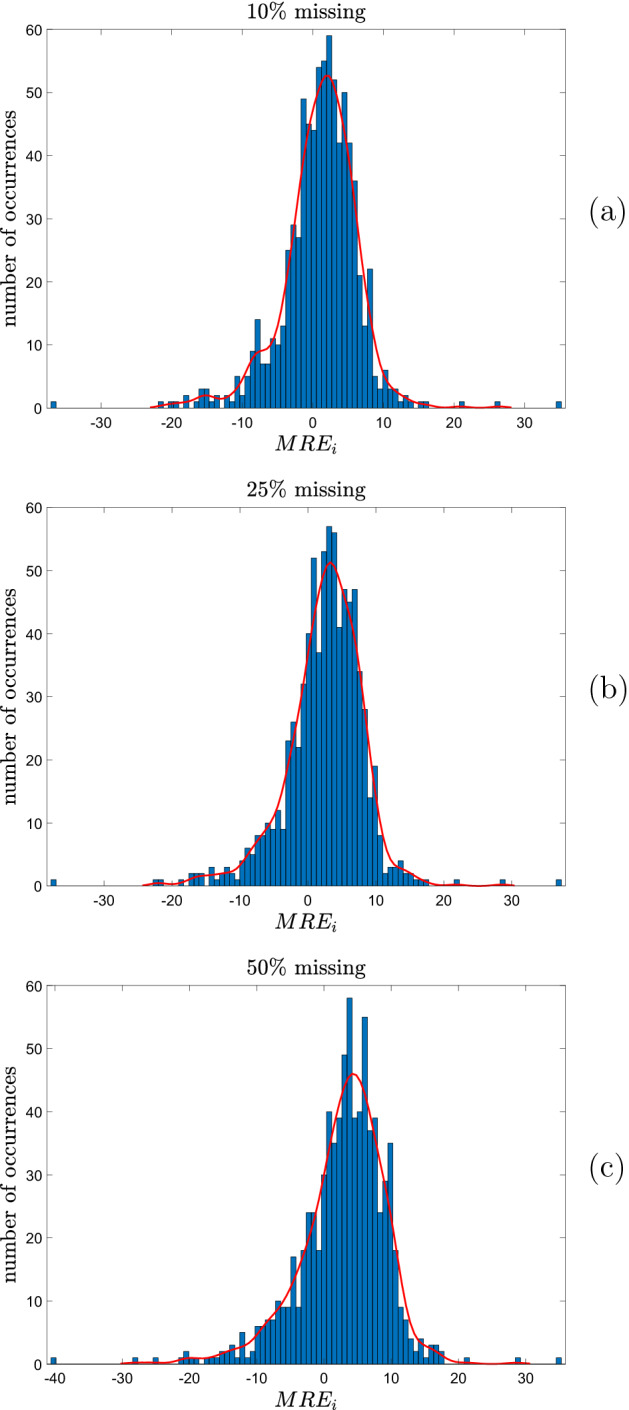
Histograms (and kernel-fitted histograms) of $MRE_i$ in the full dataset of professions, for different percentages of missing entries in the columns associated with creativity: **a** $10\%$ missing entries in the selected columns; **b** $25\%$ missing entries in the selected columns; **c** $50\%$ missing entries in the selected columns

What we find (see Fig. 6 for the histograms of the $MRE_i$ in the three cases) is that around 65% of occupations deviate positively (i.e., they have positive $MRE_i$). Moreover, deviations are concentrated in the interval [− 1%, +5%]. For instance, in the case of 10% missing entries associated with creativity, about 52% of the observations of the $MRE_i$ fall in this interval, meaning that the prediction error is typically very small (see also Table 4 for summary statistics, and Table 5 for results at the level of each major group).

Referring to the list reported in Sect. 3, the professional groups experiencing most of the positive deviations are: the second (intellectual, scientific, highly specialized professions), the third (technical professions), the sixth (artisans, specialized workers, agriculturalists) and the seventh (plant operators, machinery workers, drivers of vehicles), while the main skills explaining these results (i.e., which contribute to the summation in Eq. (2) with the largest positive terms) are: listening, human resources management and creative thinking.

The remaining professions whose predictions deviate negatively are mostly in the first (legislators, entrepreneurs, senior management), third (technical professions), sixth (artisans, specialized workers, agriculturalists), seventh (plant operators, machinery workers, drivers of vehicles) group, while the associated skills that motivate the negative differences are analytical skills and solving unexpected problems.

In summary, there is a need to reinforce competences such as solving unexpected problems, listening actively, critical sense, adaptability and service orientation, which concerns transversely all the professional groups, including technicians and associate professionals as well as service and sales workers, artisans, specialized workers and agriculturalists and, to a smaller extent, plant and machine operators and assemblers.

The most intense skill upgrading needs are observed in the high-tech manufacturing sector (chemical and pharmaceutical, electronic, energetic, engineering industry) but they are quite relevant also in the service sector when related to education, health, communication, financial activities and other services.

To address the issue above in more detail, in Table 6 we report 4 professions for each major group whose $MRE_i$ is among the largest in absolute value, and the relative skills in which they deviate the most, both positively and negatively.

**Table 4:** Summary statistics for the distribution of $MRE_i$ in the full dataset of professions, for different percentages of missing entries in the columns associated with creativity

	10% missing		25% missing		50% missing	
	# professions	%	# professions	%	# professions	%
$MRE_i$ $> 5\%$	151	18.97	264	33.17	313	39.32
$MRE_i$ $\in [-1\%;+5\%]$	419	52.64	367	46.10	308	38.69
$MRE_i$ $< -1\%$	226	28.39	165	20.73	175	21.98
*Total*	796	100	796	100	796	100

**Table 5:** Distribution of $MRE_i$ in the interval $[-1\%; +5\%]$ by major group, for different percentages of missing entries in the columns associated with creativity. The total number of professions for each major group is also reported in the table

Major groups	10% missing	25% missing	50% missing	Observations
Professions with $MRE_i$ in $[-1\%; +5\%]$	% professions	% professions	% professions	total # professions
1. Legislators, managers	64.18	49.25	37.31	67
and senior officials				
2. Intellectual, scientific and	68	48.57	37.14	175
highly specialized professionals				
3. Technicians and associate	53.14	48.75	45	160
professionals				
4. Clerical support workers	30	33.34	36.67	30
5. Service and sales workers	39.68	38.09	30.16	63
6.Artisans, specialized	44.70	45.88	39.41	170
and agricultural workers				
7. Plant and machine	47.57	48.54	41.74	103
operators and assemblers				
8. Elementary occupations	32.14	32.14	21.43	28

**Table 6:** Professions, means of the observed values of creative soft skills, $MRE_i$ (in the case of $10\%$ missing observations in the selected columns) and skills which contribute most to the positive/negative deviation

Professions	Mean true values	$MRE_{i}$ 10%	Largest pos. dev. skills	Largest neg. dev. skills
Police chiefs	82.52	7.15	ANSK, INN, CREAT	LEAST, UNDOTH, UNEX
PA managers	79.20	8.21	AD, DES	NEG, SERV, UNEX
Firm managers (manufacture)	55.05	$-$ 3.16	SERV, MRM, DES	CRIT, NEG, DMAK
Firm managers (building)	59	$-$ 1.82	SERV, ANSK, CREAT	LEAST, FRM, HRM
Cartographers	46.05	$-$ 6.16	UNDOTH, PERS, SERV	ANSK, CLASS, INN
Dietologists	57.80	$-$ 6.17	CRIT, UNDOTH, NEG	PERS, TEACH, DES
Lawyers	62.05	11.04	ANSK, SERV, CREAT	PERS, NEG, DMAK
Dialogists	51.87	11.29	PERS, FRM, HRM	AD, INN, CREAT
IT technicians	63	26.40	CRIT, TIME, HRM	LIST, LEAST, UNDOTH
Interviewers	16.50	$-$ 36.67	CRIT, AD, SERV	LIST, TIME, AD
Athletes	29	$-$ 15.22	MRM, DES, CREAT	ALEARN, NEG, DMAK
Presentators	53.84	13.60	CRIT, SERV, COMPL	DES, CREAT
Typists	28.20	$-$ 21.31	UNDOTH, PERS, COMPL	MRM
Data entry officers	35.90	15.55	ALEARN, UNDOTH, DES	TIME
Purchasing managers	49.82	6.18	CRIT, CREAT	DMAK, FRM, DES
Post office workers	34.82	6.44	UNDOTH, AD, ANSK	TIME, DES
Shop assistants	19.90	$-$ 19.87	CRIT, ALEARN, UNDOTH	LIST, LEAST, TIME
Remote sellers	35.60	20.89	TEACH, UNEX, FRM	PERS, NEG, DES
Waiters	32.31	$-$ 14.97	UNDOTH, NEG, FRM	UNEX, HRM, AD
Hairdressers	52.20	13.31	AD, NEG, TEACH	ALEARN, UNDOTH, PERS
Tilers	27.75	13.38	UNDOTH, NEG, DES	LIST, CRIT, AD
Tunnel owners	46.62	$-$ 19.33	TEACH, COMPL	UNDOTH, NEG, ANSK
Planes mechanics	45.87	$-$ 18.01	UNDOTH, NEG, SERV	AD, INN
Engravers	54.35	11.92	ALEARN, PERS, TIME	CRIT, NEG, FRM
Electrochemical plant workers	52	35.14	LEAST, UNDOTH, NEG	LIST, CRIT, AD
Machinery operators	34.36	16.18	UNEX, DMAK, CREAT	ALEARN, LEAST, SERV
Bus conductors	24	$-$ 14.64	LEAST, AD, DMAK	UNDOTH, FRM, DES
Boiler conductors	29	$-$ 15.98	ALEARN, LEAST, AD	DES, INI
Cleaning staff	25.10	$-$ 15.08	CRIT, ALEARN, AD	TIME, MRM
Forestry personnel	52.33	11.75	LIST, CRIT, UNEX	NEG, SERV, DES
Fishing/hunting personnel	65	15.58	LIST	ALEARN, UNDOTH, NEG
Industrial activity personnel	30.35	$-$ 13.78	PERS, NEG, SERV	LIST, AD, CLASS

### Results Related to Specific Economic Sectors

In this subsection, we focus our analysis on two economic sectors, the health system and the cultural industry, by applying MC to the respective occupation submatrices associated with professions belonging to each of the two sectors. These two sectors are chosen for the following reasons. In the case of the health system, we are interested to identify professions at a high risk of being automated, due to actual creativity levels smaller the ones predicted by the counterfactuals generated by matrix completion. On the one hand, the healthcare sector is a working context in which creativity is often assumed to play a limited role (at least in the cases for which routine tasks are common). On the other hand, the analysis of the cultural industry is performed to get a further validation of the method, since the creativity levels for the professions in this sector are typically expected to be higher than the ones of the professions belonging to other sectors. So, in this case, we check that the predictions of MC are consistent with this expectation.^10^

**Fig. 7:**
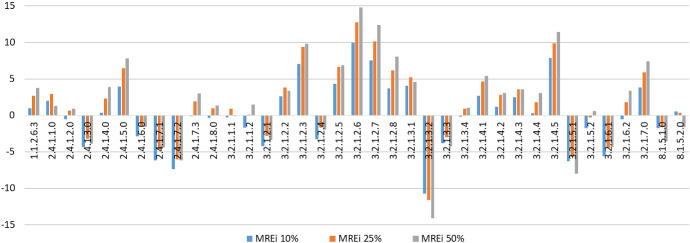
$MRE_i$ in the Health Sector-ADA 19 (ADA stands for *Area di Attività*, or *Area of Activity*), The correspondence between codes and professions is reported in Table 7 in the Appendix (color figure online)

Figure 7 shows that the creative behaviour of professions in the healthcare sector is highly heterogeneous: categories of health workers dealing with patients on a daily basis are used to interact more with people, thus the need for soft skills in general and creativity in particular is higher. This also implies that MC negative deviations (i.e., underestimates of the true creativity level) are likely to occur.

That is exactly the case for jobs like Therapists in neuropsychomotricity (3.2.1.2.5), Therapists in psychiatric rehabilitation (3.2.1.2.6), Social educators (3.2.1.2.7) and Occupational therapists (3.2.1.2.8). On the other end of the distribution we find jobs that are mainly dealing with technical diagnostic, such as Diagnostic imaging and radiotherapy specialists (2.4.1.6.0) and Biomedical laboratory assistants (3.2.1.3.2).

**Fig. 8:**
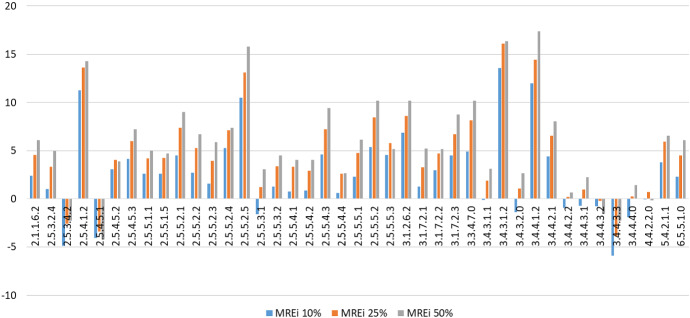
$MRE_i$ in the Cultural Industry-ADA 22 (ADA stands for *Area di Attività*, or *Area of Activity*). The correspondence between codes and professions is reported in Table 8 in the Appendix (color figure online)

In the case of the cultural sector, instead, the distribution is smoother and creativity patterns are more homogeneous among professions (see Fig. 8). In particular, the MC predictions for almost all kinds of workers in this sector deviate positively and the magnitude of these deviations is quite large.

The most creative occupations, according to the results of our analysis above, are Dialogists (2.5.4.1.2), Set designers (2.5.5.2.5), Artistic performances presenters (3.4.3.1.2) and Stage builders (3.4.4.1.2), while Painters and sculptors (2.5.5.1.1), Directors (2.5.5.2.1), Actors (2.5.5.2.2), Artistic directors (2.5.5.2.3), Scriptwriters (2.5.5.2.4) take values in the middle of the distribution.

### The Evolution of Creativity Needs

Recent developments in the MC literature extend the range of applications of this method to causal inference with panel data (Athey et al. 2021). They show that MC outperforms other synthetic control methods, as its simulations are based on real data. Thanks to the availability of the first edition of the *Survey on Occupations*, which was run in 2007, in this subsection we extend our analysis to this year, in order to assess whether our model has some predictive power over time, when the results of the MC analysis of the 2007 dataset are used to predict creativity levels observed later in 2013.

The first edition of the ICP adopted a slightly different occupation classification (*Classificazione delle Professioni 2001* or *Classification of Professions 2001*, CP2001), as this was subject to changes operated by ISTAT in 2011. Even though occupations are clustered into the same major groups in both editions of the survey, the two different classification criteria adopted do not allow to get a perfect one-to-one matching of professions at the 5-th digit occupational level. As a consequence, we reduce the rows of our two datasets (related to the years 2007 and 2013, respectively) to those professions for which there was either no change in the classification code, or a negligible change in the job description occurred. In this way, the two occupation matrices are reduced to 510 closely matching professions. Then, we apply to each of the reduced 2007 and 2013 occupation matrices the same kind of MC analysis described in the previous parts of Sect. 5 and in the Appendix. As before, the analysis is performed for each of the three percentages of missing observations in the columns associated with creative skills ($10\%$, $25\%$, $50 \%$). In particular, for each pair (*i*, *j*), where *i* denotes a profession in the reduced subset of 510 professions, and *j* one of the selected creative skills, we compare the mean MC prediction of the average importance level of skill *j* for the profession *i* based on the 2013 and 2007 datasets, respectively, i.e., we compute^11^

3 $$\begin{aligned} \Delta {\overline{predicted}}_{i,j}=\frac{1}{r_i} \sum _{r=1}^{r_i} \left( predicted_{i,j,r}^{(2013)}-predicted_{i,j,r}^{(2007)}\right) \end{aligned}$$

(again, to avoid burdening the notation, the dependence on the percentage of missing elements in the selected 25 columns is not reported in Eq. (3)).

**Fig. 9:**
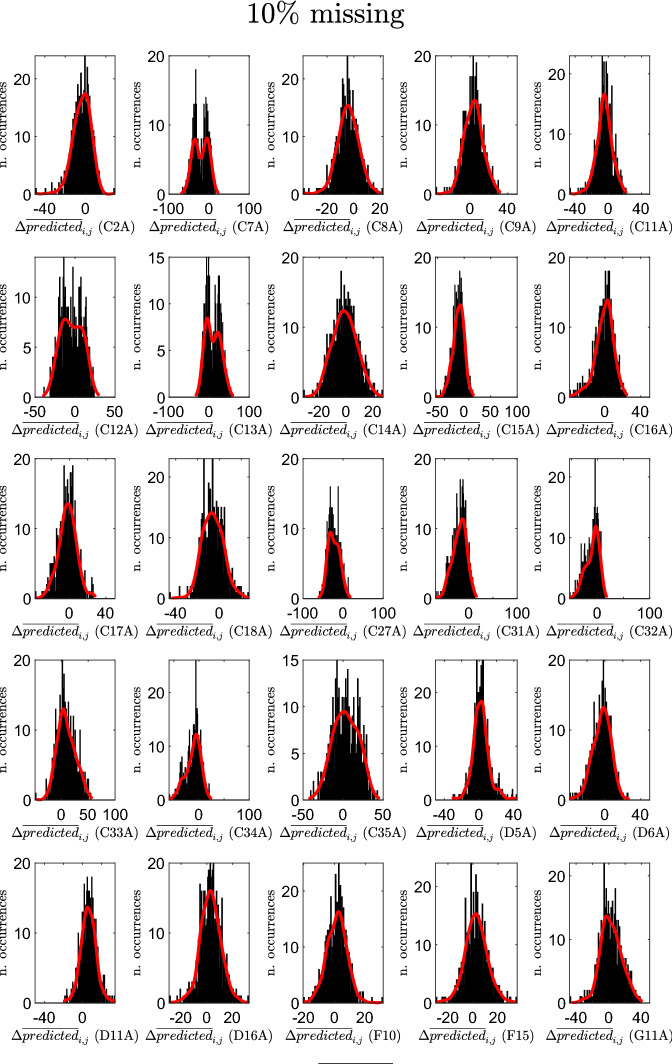
Histograms of the variations $\Delta {\overline{predicted}}_{i,j}$ for the various creative soft skills *j*: case of $10\%$ missing entries in selected columns

**Fig. 10:**
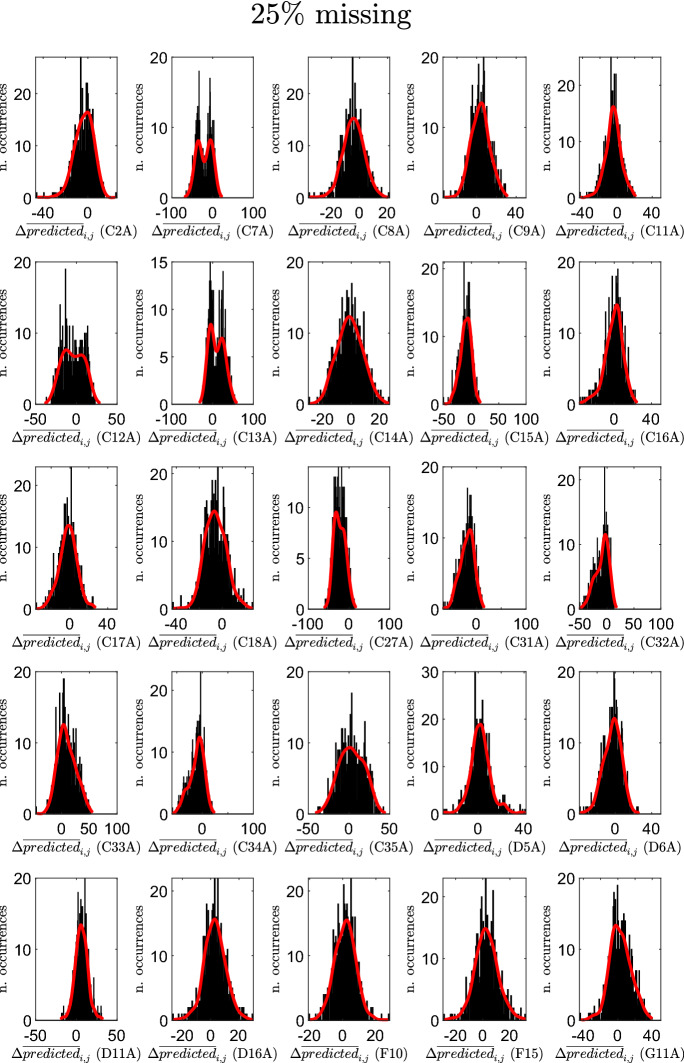
Histograms of the variations $\Delta {\overline{predicted}}_{i,j}$ for the various creative soft skills *j*: case of $25\%$ missing entries in selected columns

**Fig. 11:**
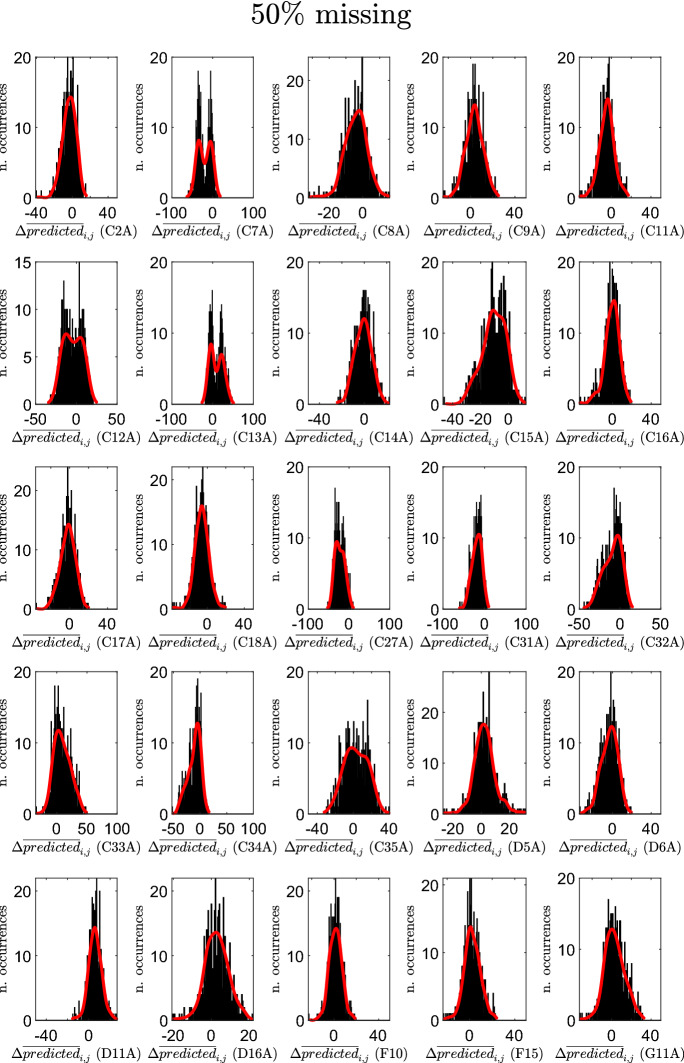
Histograms of the variations $\Delta {\overline{predicted}}_{i,j}$ for the various creative soft skills *j*: case of $50\%$ missing entries in selected columns

Figures 9, 10, and 11 report, for the three cases, and for each creative soft skill *j*, the resulting histograms of the variations $\Delta {\overline{predicted}}_{i,j}$ (in each histogram, *j* is fixed, whereas *i* varies). These figures show that the creative soft skills for which the largest positive variations are obtained are persuading, creative thinking, financial resource management and classification, while the creative soft skills with the largest negative variations are listening actively, teaching, time management and solving unexpected problems.

**Fig. 12:**
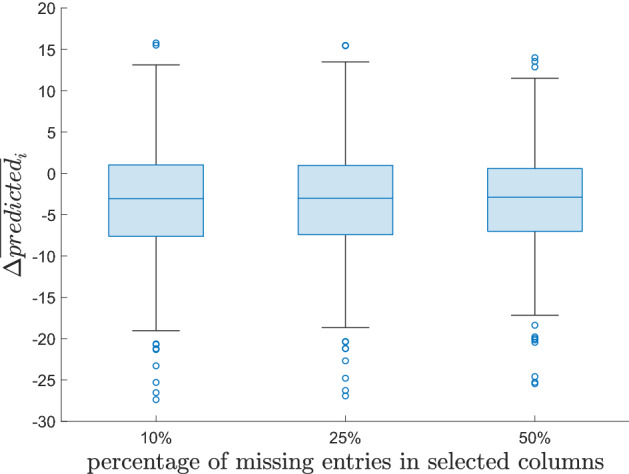
Boxplots of the variations $\Delta {\overline{predicted}}_{i}$ for the three cases considered in the analysis

As a further comparison, Fig. 12 reports the boxplots of the averages

4 $$\begin{aligned} \Delta {\overline{predicted}}_{i}=\frac{1}{25}\sum _{j \in J} \Delta {\overline{predicted}}_{i,j} \end{aligned}$$

over the creative soft skills, for the three cases considered in the analysis. The figure also shows that the [first quartile, third quartile] intervals of $\Delta {\overline{predicted}}_{i}$ are, in the three cases, $[-7.64 \%,1.00 \%]$, $[-7.51 \%,0.96 \%]$, $[-7.13 \%,0.55 \%]$, respectively. Hence, the predictions—based on the MC analysis of the reduced 2007 dataset—of the creativity levels for the professions considered in the two analyses are quite similar to the corresponding predictions based on the reduced 2013 dataset.

The professions *i* in the reduced datasets for which the average $\Delta {\overline{predicted}}_{i}$ assumes the largest negative values are: Shop assistants (5.1.2.2.0), Meter readers (8.1.1.2.0), Remote sellers (5.1.2.5.2), Biomedical laboratory technicians (3.2.1.3.2) and Plant operators in sugar refining (7.3.2.5.0). Similarly, the ones for which the average $\Delta {\overline{predicted}}_{i}$ assumes the largest positive values are: Plant operators in pottery production (7.1.3.3.1), Flame welders (6.2.1.2.0), Electric welders (6.2.1.7.0), Assemblers of composite industrial articles (7.2.7.9.0) and Hunters (6.4.5.4.0). In the period 2007–2013, the professions that experienced the largest increase in the predictions of the average importance levels of creative soft skills belong mainly to the 6-th (Artisans, specialized and agricultural workers) and 7-th (Plant and machine operators and assemblers) major groups. These are major groups characterized by a high level of routinary (manual and cognitive) tasks (Gualtieri et al. 2018). Thus, these results suggest that technological change, advances in ICTs, the advent of Industry 4.0 and the last developments in artificial intelligence, which might lead to a substitution of mechanical and repetitive jobs, are forcing these same jobs to adapt and reshape their soft skills provision.

## Final Discussion

The recent economic literature is animating a lively debate on the effects of digitalization on employment, ending up claiming that routinary jobs are more exposed to being substituted by machines (Cetrulo et al. 2020). In this framework, the existence and the magnitude of the substitution effect depend on the tasks involved in each job: in particular, routine tasks that are easier to codify are more likely to be automated, while non-routinary tasks that comprise a larger use of soft skills might be less exposed to this risk (Cirillo et al. 2021). Among subsets of soft skills, creativity is one of the hardest to be encoded, thus highly creative occupations tend to suffer less from the introduction of machines and digital systems. In this paper, we exploit similarities in the Italian occupational structure and implement a recently developed machine learning technique to predict the importance levels of creative skills employed in each occupation and to identify the creativity needs of occupations. We find that professions belonging to the major group of legislators, senior officials and managers, as well as intellectual professionals have a greater surplus of creative soft skills. Nevertheless, creativity patterns and trends are extremely heterogeneous and idiosyncratic and this means that there is no clear separation among technical and intellectual skills with respect to creative soft skills. Our study contributes to a nascent field on interdisciplinary research to predict the impact of AI on work activities and future jobs using machine learning (see also Poulakias 2021). Our results suggest that creativity gaps in the labor market are so peculiar that training might be tailored specifically for every occupation, to reduce its risk of being automated. In Italy, the policy response to the challenges posed by digitalization has been the extension of employment protection and short-time work to small firms that were not previously covered (Basso et al. 2022). Looking at the future, especially after recovering from the current Covid-19 pandemic, policy makers should take into account the possibility of better targeting the policies to sectors, occupations and firms as their patterns of sets of soft skills, creativity in particular, are highly heterogeneous between and within sectors. Skills demand in the country is increasing and changing rapidly, yet Italy is struggling to make the transition to a new digitalized economy where soft skills play a central role (OECD 2017). Making progresses will require that the country fosters both the demand and the supply of skills: for example, improving the quality of schooling that will, in time, translate into high-qualified workers, or encouraging and facilitating the transition to digital technologies for firms, so they can compete globally. In general, a wider strategy and a coordinated approach to skills policies is desirable to get Italy back on a growing path.

In our application, matrix completion has demonstrated an excellent prediction capability, making it meaningful to further analyze cases for which the actual creativity levels are significantly larger/smaller than the predicted ones, as the errors are possibly related to specific features of professions that distinguish them from other professions in the dataset. As a possible extension of our analysis, as soon as the data collected in the last edition of ICP (conducted in 2019) will be made publicly available, the method used in this article could also be applied to predict the most recent trends in the labour market, and possibly also to make a forecast analysis for the future trends after the current COVID-19 pandemic (Barbieri et al. 2022).

## Appendix A: Further Details on Matrix Completion and on Its Application to the Prediction of Creativity Levels for Different Professions

As already mentioned in the main text, in this work we adopt the following formulation for the Matrix Completion (MC) optimization problem, which was studied theoretically in Mazumder et al. (2010):

5 $$\begin{aligned} \underset{\mathbf{Z} \in {\mathbb {R}}^{m \times n}}{\mathrm{minimize}} \left( \frac{1}{2} \sum _{(i,j) \in \Omega ^{\mathrm{tr}}} \left( M_{i,j}-Z_{i,j} \right) ^2 + \uplambda \Vert \mathbf{Z}\Vert _*\right) . \end{aligned}$$

In the above, the regularization parameter controls the trade-off between the first data-fitting term and the second regularization term, which favors a small nuclear norm of the reconstructed matrix. The latter requirement is often related to getting a small rank of the obtained optimal solution $\mathbf{Z}_\uplambda ^\circ$ of the optimization problem (5),^12^ which follows by geometric arguments similar to the ones typically exploited to motivate the use of the classical LASSO (Least Absolute Shrinkage and Selection Operator) penalty term (Tibshirani 1996).

The connection between MC and singular values can be illustrated as follows. Given a nonzero matrix $\mathbf{M} \in {\mathbb {R}}^{m \times n}$, and denoting its rank by $\mathrm{rank}(\mathbf{M})$, it is well-known—by Eckart–Young theorem, see Theorem 2.1.2 in Moitra (2018)—that, for any $k \in \{1,\ldots , \mathrm{rank}(\mathbf{M})\}$, the best rank-*k* approximation of $\mathbf{M}$ according to the Frobenius norm is provided by its truncated singular value decomposition, in which one keeps only the *k*-largest singular values of $\mathbf{M}$, and zeroes all the others. So, for an effective application of MC, one needs a quite rapid decay to 0 of the singular values of the matrix $\mathbf{M}$ to be completed. This is an important necessary condition for such an effective application. However, such a condition is not sufficient when the matrix is only partially observed, because finding the singular value decomposition itself requires the knowledge of the whole matrix.

In our application of the Soft Impute algorithm to an occupation matrix, we combine the original MATLAB implementation of Soft Impute from Mazumder et al. (2010) with the MATLAB function svt.m from Li and Zhou (2017). First, a specific row (profession) is fixed. All its entries in 25 given columns related to creativity are obscured, and form the test set (their set of positions is denoted by $\Omega ^{\mathrm{test}}$). Then, one selects a percentage (respectively, $10\%$, $25\%$, $50\%$) of the rows of the matrix, excluding the given row. The entries in such rows are obscured in correspondence of the 25 given columns. These obscured entries form the validation set (their set of positions is denoted by $\Omega ^{\mathrm{val}}$). All the remaining entries of the matrix form the training set. It follows from the construction above that the training, validation, and test sets do not overlap. To avoid overfitting, we select the regularization constant $\uplambda$ by means of the following validation method. The optimization problem (5) is solved for several choices $\uplambda _k$ for $\uplambda$, exponentially distributed as $\uplambda _k=2^{k-1}$, for $k=1,\ldots ,15$. For each $\uplambda _k$, the Root Mean Square Error (RMSE) of matrix reconstruction on the validation set is defined as

6 $$\begin{aligned} RMSE_{\uplambda _k}^{\mathrm{val}}:=\sqrt{\frac{1}{|\Omega ^{\mathrm{val}}|}\sum _{(i,j) \in \Omega ^{\mathrm{val}}} \left( M_{i,j}-Z_{\uplambda _k,i,j} \right) ^2}, \end{aligned}$$

then the choice $\uplambda _k^\circ$ that minimizes $RMSE_{\uplambda _k}^{\mathrm{val}}$ for $k=1,\ldots ,15$ is obtained. Finally, the RMSE of matrix reconstruction on the test set is evaluated in correspondence of the optimal value $\uplambda _k^\circ$ as

7 $$\begin{aligned} RMSE_{\uplambda _k^\circ }^{\mathrm{test}}:=\sqrt{\frac{1}{|\Omega ^{\mathrm{test}}|}\sum _{(i,j) \in \Omega ^{\mathrm{test}}} \left( M_{i,j}-Z_{\uplambda _k^\circ ,i,j} \right) ^2}. \end{aligned}$$

The RMSE of matrix reconstruction on the training set is defined in a similar way. The whole procedure is repeated a sufficiently large number of times for each profession. This number is not the same for each profession due to the following method employed to reduce the total simulation time. Since the MC optimization problem (5) depends only on the training set, 200 random permutations of the rows of the matrix are generated. Each permutation is associated with a single training set. At the same time, it is associated with various different choices for the validation and test sets. More precisely, for each permutation, the first $h\%$ among such rows (with $h=10$, 25, 50), plus the successive row in the permutation, form a set ${\mathcal {S}}$ of rows, which is used to generate the union of the validation and test sets, by including in such union all the elements at the intersection between such rows and the selected columns. All the remaining elements of the matrix constitute the training set (one for each permutation). Then, $|{\mathcal {S}}|$ different test sets are constructed by including in each of them all the elements at the intersection between the selected columns and one specific row belonging to ${\mathcal {S}}$, in all possible $|{\mathcal {S}}|$ ways. The corresponding $|{\mathcal {S}}|$ validation sets are constructed by including in them all the elements at the intersection between the selected columns and the remaining rows of ${\mathcal {S}}$, in all possible $|{\mathcal {S}}|$ ways. As already mentioned, in all these $|{\mathcal {S}}|$ cases, the training set is always the same, so the MC optimization problem (5) is solved once for each choice of ${\mathcal {S}}$. It is also clear that, in this way, the number of times a specific row appears in ${\mathcal {S}}$ is not guaranteed to be the same for each row, because it depends on the specific set of random permutations generated. The number of such permutations (200) is selected in order to include each row in the sets ${\mathcal {S}}$ with high probability for a sufficiently large number of times. Indeed, the expected number of times this occurs is about 20, 50, 100, respectively for $h=10$, 25, 50. In this way, it is possible in principle to assess how the prediction on the test set associated with each row changes by varying the training and corresponding validation sets (see Fig. 4 in the main text for an example of this kind of analysis).

It is worth mentioning that in our application of MC to occupation matrices, all the very few missing entries of such matrices in the training set (when they are present) are replaced by zeros before running the Soft Impute algorithm. The value of the tolerance parameter of the algorithm is chosen as $\varepsilon =10^{-7}$. Moreover, when convergence of the algorithm is not achieved, in order to reduce the simulation time, the algorithm is stopped after $N^{\mathrm{it}}=50$ iterations. An additional post-processing step is included, thresholding to 0 any negative element (when present) of the completed matrices, and to 100 any element (when present) larger than 100. This is justified by the fact that the elements of the occupation matrices represent percentages. In order to avoid introducing new notation, in all the previous equations the expression $\mathbf{Z}_{\uplambda _k}$ can be also redefined so as to refer to each post-processed MC output. The results reported in the main text refer actually to such post-processed MC output.

A final remark can be made about the trade-off between the prediction capability and the biasedness of the MC method (see also the discussion on this issue presented in the Supplementary Material of Gnecco et al. 2022). According to Foucart et al. (2017) and Ma and Chen (2019), biasedness in MC depends, for instance, on how unobserved entries are selected (in our case, only entries associated with selected skills are obscured, and for the professions for which this occurs, all the entries associated with such skills are actually obscured). Nevertheless, in general biasedness can be beneficial to prediction capability, because of the trade-off between bias and variance (Hastie et al. 2009).

## Appendix B: Correspondence Between Codes and Selected Professions in Figs. 7 and 8

See Tables 7 and 8.

**Table 7:** Correspondence between codes and professions; healthcare sector, Fig. 7

Code	Profession
1.1.2.6.3	Health care directors
2.4.1.1.0	General doctors
2.4.1.2.0	Medical care specialists
2.4.1.3.0	Surgical care specialists
2.4.1.4.0	Laboratorians and clinical pathologists
2.4.1.5.0	Dentists and oral surgeons
2.4.1.6.0	Diagnostic imaging and radiotherapy specialists
2.4.1.7.1	Dietologists and hygienists
2.4.1.7.2	Social and occupational health specialists
2.4.1.7.3	Epidemiologists
2.4.1.8.0	Anaesthetists and critical care specialists
3.2.1.1.1	Nursing professions
3.2.1.1.2	Obstetrical professions
3.2.1.2.1	Podiatrists
3.2.1.2.2	Physiotherapists
3.2.1.2.3	Logopedists
3.2.1.2.4	Optometrists
3.2.1.2.5	Therapists in neuropsychomotricity
3.2.1.2.6	Therapists in psychiatric rehabilitation
3.2.1.2.7	Social educators
3.2.1.2.8	Occupational therapists
3.2.1.3.1	Audiologists
3.2.1.3.2	Biomedical laboratory assistants
3.2.1.3.3	Radiographers
3.2.1.3.4	Neurophysiopathologists
3.2.1.4.1	Orthopaedic technicians
3.2.1.4.2	Hearing care professionals
3.2.1.4.3	Dental hygienists
3.2.1.4.4	Cardiovascular pathophysiology and perfusion technicians
3.2.1.4.5	Dieticians
3.2.1.5.1	Technicians in prevention in the environment and the workplace
3.2.1.5.2	Health visitors
3.2.1.6.1	Opticians
3.2.1.6.2	Dental technicians
3.2.1.7.0	Popular medicine technicians
8.1.5.1.0	Janitors and assimilated
8.1.5.2.0	Aides and assimilated

**Table 8:** Correspondence between codes and professions; cultural sector, Fig. 8

Code	Profession
2.1.1.6.2	Paleontologists
2.5.3.2.4	Archaeologists
2.5.3.4.2	Art experts
2.5.4.1.2	Dialogists
2.5.4.5.1	Archivists
2.5.4.5.2	Librarians
2.5.4.5.3	Curators
2.5.5.1.1	Painters and sculptors
2.5.5.1.5	Conservators
2.5.5.2.1	Directors
2.5.5.2.2	Actors
2.5.5.2.3	Artistic directors
2.5.5.2.4	Screenwriters
2.5.5.2.5	Scenographers
2.5.5.3.1	Choreographers
2.5.5.3.2	Dancers
2.5.5.4.1	Composers
2.5.5.4.2	Conductors
2.5.5.4.3	Instrumentalists
2.5.5.4.4	Singers
2.5.5.5.1	Folk artists
2.5.5.5.2	Variety artists
2.5.5.5.3	Acrobats
3.1.2.6.2	Radio or TV broadcasters
3.1.7.2.1	Audio-video equipment and video shoots technicians
3.1.7.2.2	Sound technicians
3.1.7.2.3	Audio-video editors
3.3.4.7.0	Agents and representatives of artists and athletes
3.4.3.1.1	Radio or TV announcers
3.4.3.1.2	Presenters
3.4.3.2.0	Radio, television, cinema and theaters production technicians
3.4.4.1.2	Set designers
3.4.4.2.1	Museum technicians
3.4.4.2.2	Library technicians
3.4.4.3.1	Art estimators
3.4.4.3.2	Philatelic and numismatic experts
3.4.4.3.3	Calligraphy experts
3.4.4.4.0	Restoration technicians
4.4.2.2.0	Library officers
5.4.2.1.1	Cinema and theatre merchants
6.5.5.1.0	Stagehands and property workers

## Footnotes

1 As detailed in Sect. 3, each entry of the occupation matrix to which matrix completion is applied in this work represents the average importance level (expressed as a percentage between $0\%$ and $100\%$) of a skill/competence/working condition/working style for a specific worker type. As discussed in that section, a subset of the columns of that matrix is related to creativity.

2 See Hidalgo (2021) for a comparison between total factor productivity, the analysis of economic complexity, and matrix factorization, which is related to matrix completion.

3 Another possible related explanation for the emergence of “outlier” professions is that such professions may come from “small” groups of similar professions that are under-represented in the training set employed by matrix completion, whereas other “larger” groups of similar professions may be more represented in that set.

4 See the Appendix for details.

5 Nevertheless, in this regard, it is worth remarking that, according to the literature, the disruptive effects of automation on labour substitution are quite controversial (Fernández-Macías et al. 2021).

6 The International Standard Classification of Occupations (ISCO) is an International Labour Organization (ILO) classification structure having the aim of organizing information related to labour and jobs. ISCO is defined by ILO itself as a tool for classifying jobs into a properly defined set of subgroups, according to the specific tasks and duties involved in each job. The updated classification was adopted at the end of 2007, and is known in the literature as ISCO-08.

7 The relationship between some of these characteristics and creativity is discussed later (see the end of this section).

8 As already mentioned, the ICP dataset provides actually also some additional information, as the survey explores, e.g., also the average intensity level in the use of each skill, conditional on a sufficiently high importance level expressed by the participant for that skill. In order to simplify our application of matrix completion, only the average importance levels have been taken into account, ending up in a matrix of smaller size, and whose entries are also simpler to interpret.

9 Of course, the customer may still evaluate a-posteriori the quality of her/his personalized recommendation, which is produced as a by-product of the MC application.

10 Another reason for which we focused our attention on the occupation submatrices related to the health system and to the cultural industry is that they contain a much smaller number of rows (40 and 37, respectively) than, e.g., the one related to manufacturing (161). Indeed, in our application the MC optimization problem had to be solved a quite large amount of times (it was needed to solve such problem not only for different choices of the regularization parameter, but also for various choices of the partition of the occupation matrix in training/validation/test sets). This restricted the number of different additional analyses that could be practically performed. Another motivation for our choices is that it is hard to associate some of the occupations available in our dataset (which are grouped according to the 5-digit CP2011 classification) to a specific sector. Indeed, many professions are actually practised in different sectors. For instance, some of those professions that we included in Table 1 under the “other industries” label (e.g., agricultural industry, mining industry, energetic industry and service industry, such as finance, banking and insurance) might as well be practiced inside the manufacturing industry. Moreover, the same profession could be practised either in a private or in a public sector. Hence, an integration to the current dataset would be needed to extend the current analysis, e.g., to other economic sectors such as manufacturing, or to compare private/public sectors.

11 The same seed for pseudo-random number generation has been used for the simulations based on the reduced 2007 and 2013 datasets. So, for each profession *i* considered, the number $r_i$ is the same for the two analyses.
